# Case Report: Kasai Operation in Biliary Atresia After Extensive Bowel Resection

**DOI:** 10.3389/fsurg.2021.802859

**Published:** 2022-01-24

**Authors:** Szu-Han Chen, Kai-Chi Chang, Jia-Feng Wu, Huey-Ling Chen, Wen-Hsi Lin

**Affiliations:** ^1^Department of Pediatrics, National Taiwan University Hospital, Taipei, Taiwan; ^2^Department and Graduate Institute of Medical Education and Bioethics, College of Medicine, National Taiwan University, Taipei, Taiwan; ^3^Department of Surgery, National Taiwan University Hospital, Taipei, Taiwan

**Keywords:** biliary atresia, Kasai operation, malrotation, midgut volvulus, short bowel

## Abstract

Biliary atresia (BA) is a major and devastating cholestatic liver disease in infancy. The Kasai procedure is an operation to re-establish bile flow from the liver into the intestine that can prevent the young infant from progressing rapidly to cirrhosis. The standard Kasai procedure includes the removal of extrahepatic bile duct remnants and reconstruction. We report a case of BA with short bowel due to previous small intestinal volvulus. This full-term female infant received extensive small bowel resection after birth due to intestinal volvulus. The length of the residual small bowel was 55 cm with an intact ileocecal valve. Because of progressive cholestasis and clay stool, another laparotomy was performed under the diagnosis of BA on the 52 days old. After dissection of the hepatic portal area, a segment of the colon instead of intestine was used as a biliary conduit to avoid further shortening her small bowel. The patient recovered from the procedure uneventfully and the parenteral nutrition was discontinued 2 weeks later. Two episodes of cholangitis happened after discharge. She gradually resumed body weight gain and the bilirubin level returned to normal range 6 months after the operation. This unique case demonstrated successful use of this specific procedure in the patient with BA and short bowel that have never been reported in the literature.

## Introduction

Kasai operation is a standard surgical procedure for patients with BA to restore bile drainage. Timely surgery is crucial for native liver survival and the survival of patients. However, the standard Roux-en-Y loop structure could not be established in patients with a preexisting condition of short bowel. Some surgeons have attempted to modify reconstructive surgery by adjusting the Roux-en-Y loop length or modifying the intestinal conduits. We report an infant with BA who had undergone extensive bowel resection previously due to an episode of small bowel volvulus. A segment of the colon was harvested as the biliary conduit. The patient restored enteral feeding without parenteral nutrition after the procedure, and cholestasis gradually subsided as well.

## Case Description

A late-preterm (gestational age 36 weeks, birth body weight 1,896 g) female baby was admitted to the neonatal intensive care unit after birth. She was conceived by *in vitro* fertilization and her parents reported no family history of gastrointestinal or hepatic disease. The mother of the patient received regular prenatal examinations and fetal bowel dilation was noted on level II ultrasound. In a series of follow-ups, bowel dilation progressed and intrauterine growth restriction was noted.

After birth, a distended abdomen was noted upon physical examination of the patient, and much bile content was drawn from the orogastric tube. The patient also had elevated C-reactive protein levels (12.2 mg/dl). The abdominal ultrasonography of the patient showed many ascites and was positive for the whirlpool sign. Midgut volvulus was suspected and the patient received emergency exploratory laparotomy. The bowel was found to be twisted clockwise along the mesentery axis for at least 720°, causing long segmental ischemia. The whole bowel was examined after untwisting the volvulus. No evidence of intestinal atresia or meconium impaction was noted. The ligament of Treitz lies to the left side and the cecum was placed in the left upper abdomen, so the diagnosis of incomplete rotation with midgut volvulus was confirmed. The surgeon performed detorsion on the volvulus and created a proximal jejunostomy. In the second-look exploratory laparotomy 2 days later, the surgeon resected the necrotic segment while preserving the ileocecal valve. The remaining small bowel length was 55 cm. Gastrointestina (GI) priming was initiated 5 days after the operation and she tolerated full feeding at 18 days old. Jejunostomy closure was performed at 26 days old. Parenteral nutrition was discontinued at 1 month old, but she gained weight poorly under human milk and semi-elemental infant formula feeding.

**Figure 1:**
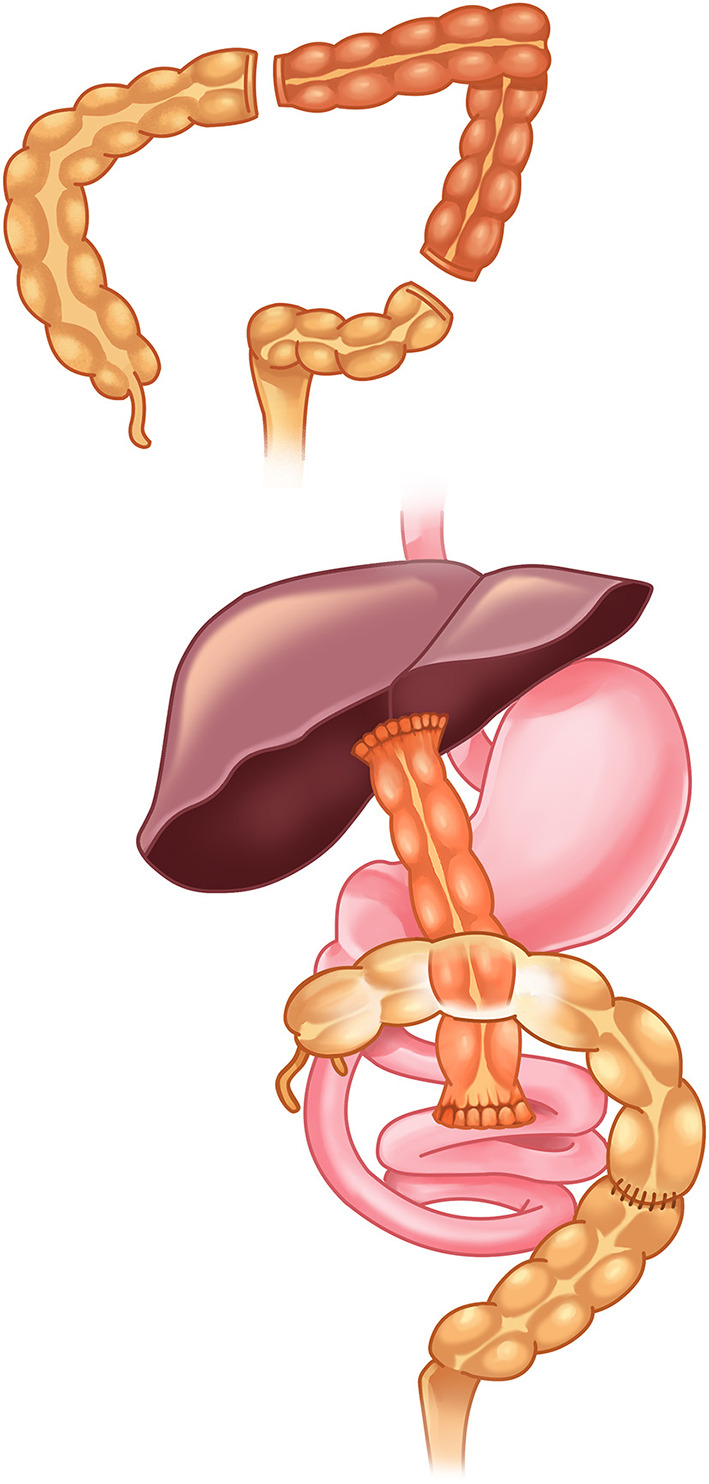
A segment of the colon was used as a biliary conduit. End-to-side colojejunostomy was performed at 10 cm from the ligament of Treitz.

The patient passed clay-colored stool and was observed to be jaundiced in the following days. Laboratory data showed elevated total bilirubin (8.5 mg/dl), direct bilirubin (5.5 mg/dl), and gamma-glutamyl transferase (364 U/l) levels. Abdominal ultrasonography exhibited a visible gallbladder, with no contraction after feeding and suspected fibrotic change at the periportal area. Hepatobiliary iminodiacetic acid scan revealed no excretion from the liver into the bowel. An intraoperative cholangiogram at 52 days old confirmed the diagnosis of biliary atresia while Kasai operation was performed. Because the length of the small intestine was 55 cm and the colon was 40 cm, we harvested 20 cm of the transverse to descending colon for the biliary conduit. After dissecting the portal area, we harvested 20 cm of the transverse to descending colon as a biliary conduit. The splenic flexure was taken down by dividing the splenocolic ligament. A 20-cm segment of the colon was transected and retracted medially with the preservation of blood supply from the mesocolon artery. Proximal and distal segments of the colon were retracted laterally and end-to-end anastomosis was performed. The conduit colon was pulled to retrocolic after the creation of a mesocolic canal. The end-to-end hepatoportocolostomy and end-to-side colojejunostomy were performed at a 10 cm site from the ligament of Treitz (Figure 1). A liver biopsy was performed during the Kasai operation, and the pathology revealed severe intrahepatic cholestasis with few bile plugs in bile ducts. There were ductular reactions, portal fibrous expansion, and focal extramedullary hematopoiesis, but no obvious hepatitis, giant cell formation, or bridging fibrosis.

**Figure 2:**
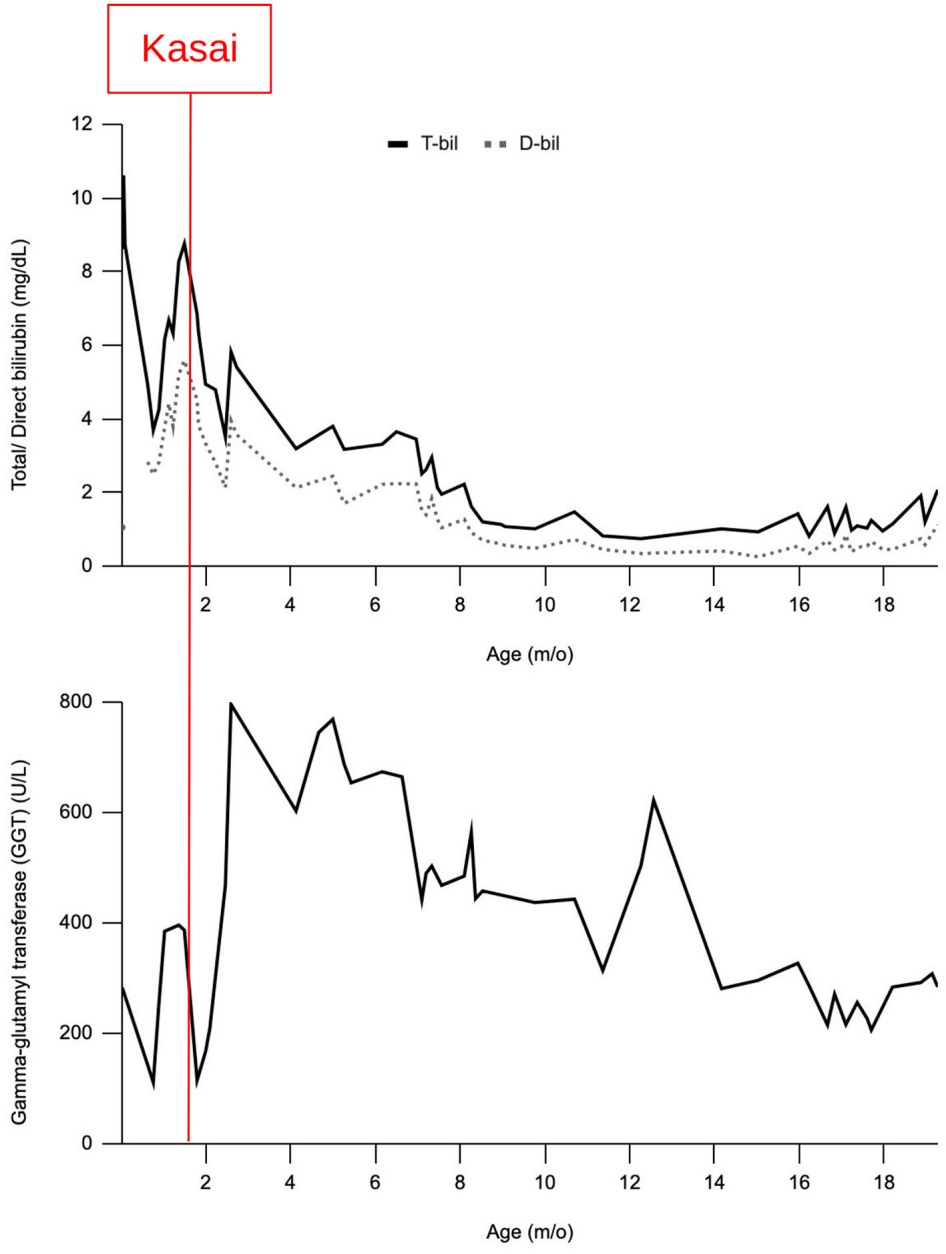
Serum biochemistry [Gamma-glutamyl transferase (GGT)/total and direct bilirubin] from birth to the latest follow-up. Operation day was marked with the red line.

**Figure 3:**
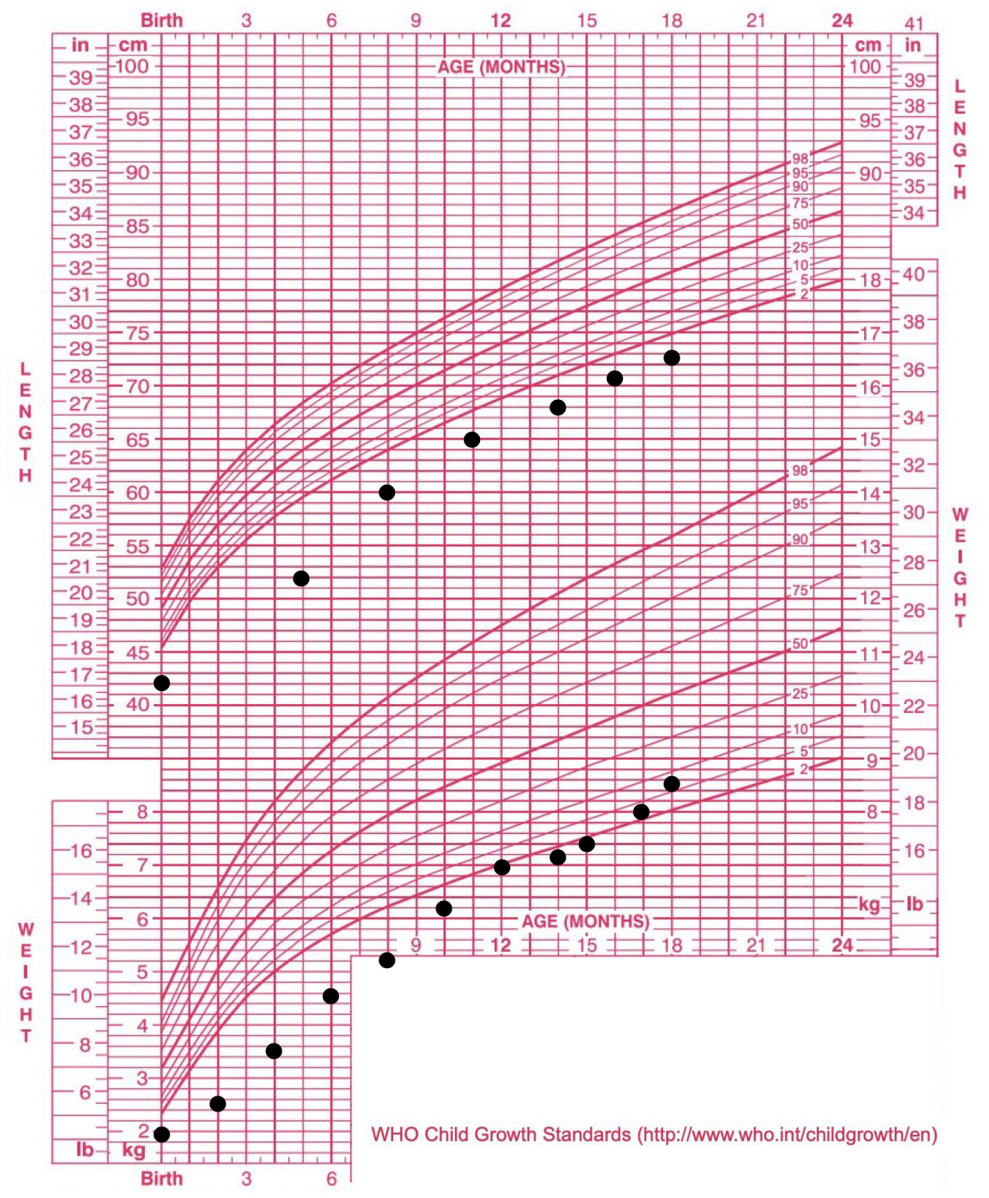
Growth curve of the patient. Significant body weight percentile catch-up was observed, as she moved from lower than the 3rd percentile to the 3–10th percentile without extra parenteral nutrition.

The patient tolerated full enteral nutrition 14 days after the operation. The stool color was greenish. The patient had no bowel motility problems in the following clinical course. There was no ascending biliary tract infection during the hospital stay. She had gained weight steadily and was discharged at 2 months and 29 days old. Her bilirubin level returned to normal at 8 months old. Three episodes of admission for cholangitis were documented for our patient. The patient has been under regular follow-up for 1.4 years now and has maintained appropriate liver function (Figure 2) and stable growth (Figure 3). She now tolerates a full diet requiring no extra parenteral nutrition and passes stool of pasty consistency 6–8 times per day.

## Discussion

Both the intestinal volvulus and BA are major surgical diseases in infants. A retrospective multicenter study reported a 0.3% incidence of intestinal malrotation in 851 patients with BA (1). Malrotation with midgut volvulus is a pediatric surgical emergency, and resultant short bowel owing to the compromised blood supply to the midgut is one of its most severe complications. It is rare but clearly possible that a patient with extensive bowel resection is subsequently diagnosed with BA. Other causes of BA with short bowel length are complications due to meconium peritonitis or intestinal atresia (2). Biliary atresia splenic malformation (BASM) is a distinct subgroup, in which malrotation is a common associated condition (3). Our patient has situs solitus without macroscopic splenic abnormality or structural cardiac anomalies, so the diagnosis of BASM was less likely.

The standard Kasai operation is performed with the creation of an enterostomy at about 10 cm distal to the ligament of Treitz to form the double-Y hepatic portojejunostomy. The distal cut is brought up to the porta hepatis to make an anastomosis to the cut surface of the fibrous mass at porta hepatis. The proximal cut is then brought to the side of the jejunum about 45 cm distal to the ligament of Treitz, where an end-to-side anastomosis is made to form the Roux-en-Y structure (4). In patients who received extensive bowel resection, the decision whether to save the small bowel to serve as the digestive tract or to make a Roux-en-Y loop long enough to prevent ascending cholangitis is difficult (5). Patients with short bowel may suffer complications such as total parenteral nutrition (TPN) associated cholestasis or central line infection, making clinical management extremely difficult.

Some surgeons have attempted to modify reconstructive surgery by adjusting Roux-en-Y loop length. A prospective randomized controlled trial reported an individualized short (13–20 cm) Roux-en-Y loop delivers a comparable antireflux effect when compared with the standard (30–40 cm) Roux-en-Y loop in Kasai operations for infants with Ohi Type III biliary atresia (6). Modifications of intestinal conduits have also been reported, including constructing hepatic portocholecystostomies (gallbladder Kasai) or appendico-duodenostomies. But none has proven entirely satisfactory in terms of eliminating cholangitis. A retrospective study of 97 cases that underwent gallbladder Kasai from a single institution reported 46% of the patients to be jaundice free with their native liver after 5 years of follow-up (7).

If our patient underwent standard Kasai operation, the length of the small bowel will be greatly reduced, which might result in the patient being dependent on parenteral nutrition. Gallbladder Kasai was not feasible in this patient because an intraoperative cholangiogram showed her distal extrahepatic bile duct was not patent. We chose to preserve the small intestines by harvesting a segment of the colon for the bile-draining conduit. The patient was able to restore full enteral feeding without parenteral nutrition.

This case demonstrated a surgical modification in Kasai operation by using a colon segment as bile drainage conduit in a patient with biliary atresia and extensive small bowel resection. The proposed procedure could restore bile drainage, while also preserving the maximal length of the small bowel. The limitation of this procedure is that shortened colon increased stool passage frequency and slow transit of colon conduit significantly increased the occurrence of ascending cholangitis. Long-term follow-up is required as ascending infection is still possible considering the limited length of the afferent loop. Furthermore, bacterial overgrowth in the small bowel in the patient with short bowel may be another risk factor of ascending cholangitis.

## Data Availability Statement

The original contributions presented in the study are included in the article/supplementary material, further inquiries can be directed to the corresponding author.

## Ethics Statement

Written informed consent was obtained from the relevant minor's legal guardian/next of kin for the publication of any potentially identifiable images or data included in this article.

## Author Contributions

S-HC and H-LC analyzed and interpreted the data. S-HC, K-CC, J-FW and H-LC participated in acquisition of data. J-FW and W-HL worked in conception and design of study. S-HC, J-FW, H-LC, and W-HL drafted manuscript and critical revision. All authors substantially contributed to the approval of final version of manuscript.

## Conflict of Interest

The authors declare that the research was conducted in the absence of any commercial or financial relationships that could be construed as a potential conflict of interest.

## Publisher's Note

All claims expressed in this article are solely those of the authors and do not necessarily represent those of their affiliated organizations, or those of the publisher, the editors and the reviewers. Any product that may be evaluated in this article, or claim that may be made by its manufacturer, is not guaranteed or endorsed by the publisher.
